# Therapeutic effect of intravenous sodium thiosulfate for uremic pruritus in hemodialysis patients

**DOI:** 10.1080/0886022X.2020.1822867

**Published:** 2020-09-24

**Authors:** Yu-Huan Song, Si-Yang Wang, Jia-Hui Lang, Yue-Fei Xiao, Guang-Yan Cai, Xiang-Mei Chen

**Affiliations:** a Department of Nephrology, Aerospace Center Hospital, Peking University Aerospace School of Clinical Medicine, Beijing, China; b Department of Nephrology, Chinese PLA General Hospital, Chinese PLA Institute of Nephrology, State Key Laboratory of Kidney Diseases, National Clinical Research Center for Kidney Diseases, Beijing, China; c Department of Nephrology, Aerospace Center Hospital Chengde Branch, Chengde, China

**Keywords:** Sodium thiosulfate, pruritus, dialysis, visual analog scale, pruritus score

## Abstract

**Objectives:**

This study aimed to compare the efficacy of intravenous sodium thiosulfate (IV STS) with that of loratadine in the treatment of uremic pruritus in hemodialysis (HD) patients.

**Methods:**

This retrospective study included 44 HD patients with pruritus aged over 18 years between June 2018 and January 2020 at the Aerospace Center Hospital of China. Twenty-four HD patients received 3.2 g IV STS treatment three times per week at the end of each HD session for 8 weeks. Twenty HD patients received loratadine (10 mg/day) for 8 weeks. Pruritus intensity was measured using a visual analog scale (VAS) and the detailed pruritus score (DPS) at three time points. The safety of STS was evaluated according to adverse event symptoms and biological variable changes.

**Results:**

There was no significant difference between the STS and loratadine groups in age, sex, characteristics of pruritus, or other clinical variables before treatment. After 8 weeks of treatment, the VAS score (7.07 ± 2.56 and 2.67 ± 2.01) and DPS (30.72 ± 4.81 and 8.04 ± 2.86) decreased significantly in the STS group (*p* < 0.05). The mean decrease in VAS score (6.89 ± 1.98 and 6.34 ± 2.35) and DPS (28.90 ± 3.24 and 26.92 ± 2.41) in the loratadine group was not statistically significant (*p* > 0.05). There were no morbidities or mortalities associated with the use of either drug. All biological variables remained stable after therapy.

**Conclusions:**

STS can improve uremic pruritus in HD patients. However, literature on the subject remains lacking. Close monitoring for adverse effects is advised.

## Background

Uremia affects almost all body organs, including the skin, through different mechanisms, such as biochemical, vascular, neurologic, immunologic, hematologic, electrolyte-related, and volume imbalance-mediated mechanisms [1]. Pruritus is one of the common symptoms in uremic patients undergoing hemodialysis (HD) [2]. Patients with more severe pruritus tended to have higher serum C-reactive protein (CRP) levels and lower serum albumin levels [3]. Mortality was found to be significantly higher in patients with pruritus than in those with unaffected skin [4]. However, large numbers of uremic patients with pruritus do not receive effective treatment [5].

Sodium thiosulfate (STS) has been used to treat calcific uremic arteriolopathy (CUA) in HD patients [6]. CUA, also referred to as calciphylaxis, is a thrombotic disorder of the skin and subcutaneous tissue [7,8]. Reported patient symptoms included pain, skin lesions, and pruritus [9]. STS is one of the cornerstones of calciphylaxis therapy and has been used for over 10 years for this indication, although this use remains off-label [9]. It also decreases metastatic calcification in patients with chronic kidney disease (CKD) [10]. The recommended dosage for the treatment of CUA is 12.5 g to 25 g, three times per week at the end of each HD session for 6 weeks to 34 months [11–13]. Several Chinese studies reported that STS had a nonspecific antiallergic and antipruritic effect, which can be helpful with regard to relieving asthma and atopic dermatitis [14,15]. It acts as a potent antioxidant by regenerating glutathione and prompting endothelial nitric oxide production through the action of hydrogen sulfide, thereby increasing tissue blood flow and oxygenation [16,17]. This multifactorial activity is perhaps responsible for the response in pruritus patients.

Although the possible mechanism remains unclear, the effects of STS on uremic pruritus have been reported in few Chinese studies [18,19]. This study compared the efficacy and safety of intravenous sodium thiosulfate (IV STS) with those of oral loratadine in HD patients with uremic pruritus.

## Methods

A total of 24 maintenance HD patients who complained of pruritus and were treated with STS between June 2018 and January 2020 at the Aerospace Center Hospital of China were included in this retrospective study. Pruritus was defined as an unpleasant sensation leading to scratching. We selected another 20 maintenance HD patients with pruritus who used oral loratadine (10 mg/day) as the control group. The same type of dialyzer was used in the two groups of patients throughout the study period.

The inclusion criteria were as follows: patients with pruritus who were older than 18 years and were on maintenance HD three times a week for at least 3 months; patients who did not receive any drug for the treatment of uremic pruritus before STS or loratadine; and patients who finished the VAS (visual analog scale) score and DPS (detailed pruritus score) questionnaires at three time points (at the start and the end of the therapy, 1 week after the cessation of drug therapy). The exclusion criteria were as follows: patients with other pruritus-related skin diseases, as diagnosed by an experienced dermatologist and patients who discontinued treatment before 8 weeks. To enable the investigation of the association of STS treatment with inflammation in HD patients, those who had autoimmune disorders, used immunomodulatory drugs, had infections, were malnourished or had other diseases affecting the level of high-sensitivity CRP (hs-CRP) were also excluded.

The dosage of IV STS treatment was 3.2 g administered in 20 ml of normal saline during the last 5 min of each HD session three times per week for 8 weeks. The dosage of loratadine was 10 mg/day for 8 weeks. The study was approved by the ethics committee of the Aerospace Center Hospital of China and written informed consent was obtained from the patients.

The data collected included age, sex, dialysis vintage, intradialytic weight gain, vascular access type, and the levels of hemoglobin, albumin, serum creatinine, cholesterol, triglyceride, potassium, sodium, total serum calcium, phosphorus, bicarbonate, parathyroid hormone (PTH), alkaline phosphate, hs-CRP, and serum ferritin before starting and at the end of the therapy. All venous blood samples were taken immediately before a HD session.

To assess the severity of pruritus, we used a VAS and the DPS proposed by Duo (Table 1), based on a combined score for the severity and distribution of pruritus and sleep disturbance [20]. The sleep disturbance and severity/distribution scores were added to calculate the patient’s final DPS at the start and the end of the therapy. The VAS was a 10-cm vertical line marked at 0 (no pruritus or sleep disturbance) and 10 (maximum intensity of these disorders) and was scored to the nearest millimeter. The method of marking the pruritus scale was explained to each patient by the corresponding physician, while the scores were recorded by the patients themselves without the nephrologist present. The duration of sleep and time to fall asleep were also recorded. A follow-up visit was scheduled for 1 week after the cessation of STS therapy to investigate whether STS should be used continuously or intermittently. We also used the VAS and DPS to assess the severity of pruritus. All the scores were measured at three time points.

**Table 1.** Detailed Duo pruritus score system (0–40 score).

Scores	Extent of scratching	Distribution range	Frequency of attacks	Sleep disturbances
1	Light itching without scratching	One part	When the short attack (<10 min per time) occurs four times or the long attack (>10 min per time) occurs 1 time, 1 point is recorded, up to 5 points	Waking up for itching every time is recorded as 1 point, up to 14 points
2	Scratching without hurting skin	Scattering in different parts		
3	Inching on without relieving after scratching	The whole body		
4	Inching on after scratching with hurting skin	–		
5	Dysphoria	–		

Scores are recorded separately in the morning and afternoon, according to extent of scratching, distribution range, and frequency of attacks, in addition, 14 points from sleep disturbance. Thus, the highest score may be (5 + 3 + 5) × 2 + 14 = 40 during the 24 h.

The safety of STS was evaluated based on adverse event symptoms (such as poor appetite, anorexia, nausea, vomiting, dizziness, and intradialytic hypotension) and changes in serum bicarbonate, anion gap, sodium, calcium, phosphorus, and PTH after administration of STS.

### Statistical analysis

All continuous variables are reported as the means ± standard deviations and were compared using an independent sample *t*-test. The chi-square test was used for categorical variables, and multivariable repeated-measure analysis of variance was used for the overall comparison of the STS and loratadine groups during the study. All statistical tests were performed using SPSS (version 22, SPSS, Chicago, IL, USA). A *p* value less than 0.05 was considered indicative of significance.

## Results

A total of 44 maintenance HD patients (32 males and 12 females; mean age: 63.88 ± 6.54 years; range: 33–86 years; HD vintage: 43.02 ± 25.31 months) fulfilled the inclusion criteria. The characteristics of the STS and loratadine groups before treatment are presented in Table 2. There were no significant differences between the two groups with regard to age, sex, dialysis vintage, intradialytic weight gain, or the levels of hemoglobin, albumin, serum creatinine, cholesterol, triglyceride, potassium, sodium, total serum calcium, phosphorus, bicarbonate, PTH, alkaline phosphate, hs-CRP, and serum ferritin before treatment.

**Table 2.** Patient demographic and laboratory data of sodium thiosulfate and loratadine groups before treatment.

Variable	Sodium thiosulfate	Loratadine	*p*
Age, years	66.80 ± 15.32	64.81 ± 16.44	0.54
Male	18/24	14/20	0.51
Diabetes mellitus	12/24	12/20	0.96
Body weight, kg	70.52 ± 10.79	68.87 ± 11.33	0.28
Arteriovenous fistula	17/24	15/20	0.51
Dialysis vintage, months	43.02 ± 25.31	42.59 ± 23.02	0.35
Intradialytic weight gain, kg	3.07 ± 0.38	2.96 ± 0.42	0.32
Serum urea nitrogen, mmol/L	21.18 ± 4.91	23.04 ± 4.67	0.64
Serum creatinine, µmol/L	897.62 ± 369.29	860.86 ± 382.14	0.92
Total serum calcium, mmol/L	2.12 ± 0.14	2.08 ± 0.31	0.23
Serum phosphorus, mmol/L	2.01 ± 0.58	2.13 ± 0.46	0.99
Calcium–phosphorus product	4.39 ± 1.14	4.28 ± 3.61	0.93
Parathyroid hormone, pg/ml	245.33 ± 143.27	241.31 ± 160.89	0.30
Hemoglobin, g/L	117.42 ± 11.29	112.93 ± 23.84	0.84
Albumin, g/L	37.52 ± 2.27	36.60 ± 2.02	0.26
Triglyceride, mmol/L	2.41 ± 1.29	2.21 ± 1.06	0.09
Cholesterol, mmol/L	3.72 ± 0.81	3.41 ± 1.64	0.96
Kt/V	1.24 ± 0.13	1.21 ± 0.22	0.25
Serum ferritin, µmol/L	84.31 ± 78.08	105.0 ± 102.6	0.09
Visual analog scale score	7.07 ± 2.56	6.94 ± 1.98	0.78
Detailed pruritus scale score	30.72 ± 4.81	28.90 ± 3.24	0.20
High-sensitivity C-reactive protein, mg/L	5.04 ± 2.41	4.99 ± 3.21	0.73

**Figure 1.**
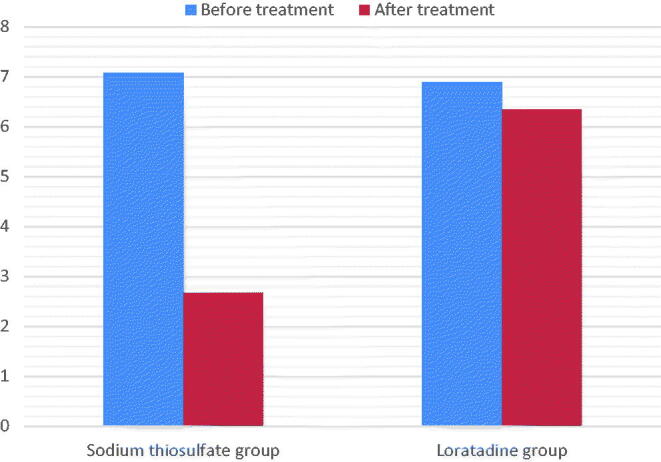
Intensity of pruritus as assessed by visual analog scale from begin to end of treatment. Comparison between sodium thiosulfate and loratadine effects.

**Figure 2.**
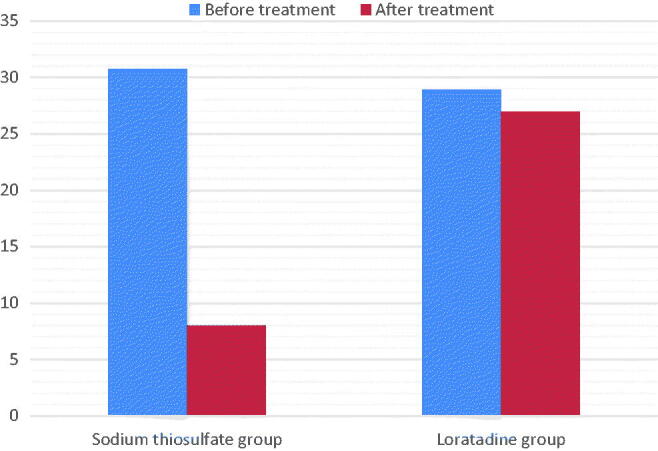
Intensity of pruritus as assessed by detailed pruritus scores from begin to end of treatment. Comparison between sodium thiosulfate and loratadine effects.

In the STS group, the age of HD patients with uremic pruritus was 66.80 ± 15.32 years, 18/24 were male, 17/24 had arteriovenous fistula (AVF), and 12/24 suffered from diabetic nephropathy. Table 3 and Figures 1 and 2 show the characteristics of pruritus before and after treatment in the STS and loratadine groups. Both the VAS score (7.07 ± 2.56 and 2.67 ± 2.01) and DPS (30.72 ± 4.81 and 8.04 ± 2.86) decreased significantly after STS treatment (*p* < 0.05). The mean decrease in the VAS score (6.89 ± 1.98 and 6.34 ± 2.35) and DPS (28.90 ± 3.24 and 26.92 ± 2.41) in the loratadine group was not statistically significant (*p* > 0.05). The VAS scores and DPSs were not significantly different between 1 week after the cessation of the drug and immediately after STS treatment (Table 4).

**Table 3.** Comparison of measured parameters before and after treatment between sodium thiosulfate and loratadine groups.

Variable	Sodium thiosulfate	Loratadine	*p*
Visual analog scale			
Before treatment	7.07 ± 2.56	6.89 ± 1.98	0.36
After treatment	2.67 ± 2.01	6.34 ± 2.35	0.003*
Detailed pruritus score			
Before treatment	30.72 ± 4.81	28.90 ± 3.24	0.56
After treatment	8.04 ± 2.86	26.92 ± 2.41	0.012*
High-sensitivity C-reactive protein, μg/mL			
Before treatment	5.04 ± 2.41	4.99 ± 3.21	0.78
After treatment	4.95 ± 2.62	4.88 ± 3.74	0.29

* *p* < 0.05.

**Table 4.** The comparison of measured parameters after treatment of STS and 1 week after the stoppage of the drug (*p* > 0.05).

Variable	Immediately after STS therapy	1 week after the cessation of STS therapy	*p*
Visual analog scale	2.67 ± 2.01	2.72 ± 1.78	0.54
Detailed pruritus score	8.04 ± 2.86	8.11 ± 1.90	0.79

Adverse effects were mild and self-limited. No adverse reactions were observed in the loratadine group. Only one patient developed temporary palpitations after a few minutes of STS treatment, and they resolved spontaneously. There were no morbidities or mortalities associated with the use of either drug. There was no significant change in the hs-CRP level after treatment in either group. Table 5 summarizes the trends in laboratory variables in the patients in the two groups. Compared with pretreatment levels, the levels of serum bicarbonate, anion gap, potassium, sodium, calcium, phosphorus, and PTH remained stable during therapy.

**Table 5.** Laboratory measures for patients who completed sodium thiosulfate or loratadine therapy.

Variable	Before treatment	After treatment	*p*
Serum sodium, mmol/L			
Sodium thiosulfate group	139.14 ± 82.49	138.09 ± 73.18	0.54
Loratadine group	138.93 ± 69.22	139.72 ± 92.46	0.38
Serum potassium, mmol/L			
Sodium thiosulfate group	4.16 ± 0.62	4.03 ± 0.70	0.68
Loratadine group	4.27 ± 0.74	4.36 ± 0.62	0.77
Serum calcium, mmol/L			
Sodium thiosulfate group	2.12 ± 0.14	2.20 ± 0.20	0.73
Loratadine group	2.18 ± 0.31	2.22 ± 0.37	0.45
Serum phosphorus, mmol/L			
Sodium thiosulfate group	2.01 ± 0.58	2.16 ± 0.39	0.96
Loratadine group	2.13 ± 0.46	2.21 ± 0.32	0.65
Parathyroid hormone, pg/ml			
Sodium thiosulfate group	245.33 ± 143.27	239.23 ± 143.67	0.18
Loratadine group	261.31 ± 160.89	245.33 ± 143.27	0.09
Serum bicarbonate, mmol/L			
Sodium thiosulfate group	20.88 ± 3.01	20.28 ± 3.22	0.46
Loratadine group	21.04 ± 4.15	20.53 ± 2.01	0.57
Serum anion gap, mmol/L			
Sodium thiosulfate group	16.22 ± 3.21	16.81 ± 2.91	0.39
Loratadine group	15.94 ± 2.89	16.02 ± 6.39	0.17

## Discussion

Skin disorders associated with HD can markedly affect patients’ quality of life and can negatively impact their mental and physical health [21]. CUA is triggered by an imbalance in the promoters and inhibitors of vascular calcification caused by the inflammatory changes that occur in uremia [22]. STS has been shown to improve skin lesions caused by calciphylaxis [23,24]. Uremic pruritus is also considered to be an inflammatory systemic disease rather than a local skin disorder [25]. Pruritus remains a distressing problem for HD patients, causing serious discomfort and skin damage, and it is often associated with sleeping problems, depression, and reduced quality of life [5,26,27]. It is thus important to consider providing timely treatment to patients with uremic pruritus to improve their quality of life. This study found that IV STS can effectively relieve uremic pruritus in HD patients, and no significant adverse reactions were observed.

Uremic pruritus may be due to an allergy to the tubing, dialyzer, or other elements associated with dialysis [2,28]. The literature has shown associations between demographic and clinical characteristics of patients and the severity of pruritus, such as aluminum level [29], secondary hyperparathyroidism, elevated blood urea nitrogen level [30], hypercalcemia and hyperphosphatemia [31], the duration of dialysis [32], low Kt/V and sex. For example, males had 1.5-fold greater adjusted odds of having moderate or severe pruritus than females [33]. HD patients with AVF are less likely to develop pruritus [34]. Our study results were consistent with these previous findings. Comorbid conditions such as diabetes mellitus, cardiovascular disease, hypertension, and neurological disease were found to be associated with pruritus in CKD patients [33,35]. Our study showed no association between pruritus and hs-CRP levels, but such an association was reported in several studies [36].

Current pharmacological therapies for CKD-associated pruritus include antihistamines, gabapentin, pregabalin, a mast cell stabilizer, and nalbuphine [37–39]. Fifty-seven percent of medical directors used oral antihistamines for the first-line treatment of chronic pruritus despite their low efficacy [40]. STS has a small molecular weight and a serum half-life of 15 min in patients with normal renal function, but the exact mechanism of action of STS in HD patients remains poorly understood, which makes the standardization and management of STS therapy challenging [41]. STS might act by producing a salt of thiosulfate of calcium (S_2_O_3_Ca), which is extremely soluble and can be removed by dialysis [12]. Thiosulfate salts of calcium have a 250- to 100 000-fold higher solubility than other calcium salts, such as those of phosphate and oxalate [42]. In this study, uremic pruritus was ameliorated by treatment with STS in HD patients. This relief was noted in some patients within the first few days after the initiation of treatment. The mechanism by which STS relieves calciphylaxis might be related to its antioxidant properties. By restoring endothelial function, STS can enhance endothelial nitric oxide production, promote vasodilation, and reduce pain [43]. Whether STS relieves pruritus through the same mechanism needs further study.

STS induces calcium removal through chelation, and the dysregulation of calcium and phosphorous metabolism and hyperparathyroidism are clearly risk factors for uremic pruritus [2,44]. However, our observations showed that the calcium and phosphate values did not change significantly after therapy. These results are in accordance with the findings of two other studies [44,45]. There was a quick decrease in the level of ionized calcium 15 min after the administration of STS but no subsequent significant decrease in calcium. STS also exhibits antioxidative and vasodilative properties [46], which might contribute to the rapid resolution of uremic pruritus symptoms.

There is no consensus on the duration of treatment with STS. The duration of therapy has reportedly been from 2 to 8 months [47]. Most patients are treated with STS for less than 3 months [48]. Recent systematic reviews have reported a significantly lower overall mortality rate in patients receiving STS for the treatment of uremic calciphylaxis than in those receiving conventional treatment [10]. There remains a need for a more robust evidence of the efficacy of this treatment.

Similar to other reports [49], the compliance of the patients in the STS group in our study was good. STS can cause side effects, such as severe but transient nausea and vomiting [50]. Mild hypernatremia, hypokalemia, or hypocalcemia (not significant) or a mild increase in the corrected QT interval (not significant) might also be side effects [51]. Another study demonstrated that neither potassium nor sodium accumulated in the circulating blood when STS was infused in conjunction with HD [52]. Case reports have described an association of STS therapy with an increased anion gap. Moreover, severe, life-threatening acidosis out of proportion with the expected acid load due to STS may occur [53]. The mechanism is unknown but has been postulated to involve the metabolism of STS to hydrogen sulfide and the oxidation of thiosulfate by the liver or intestinal bacteria to sulfate, which may be enhanced in patients with end-stage renal disease (ESRD) due to their reduced medication clearance capacity [17]. Decreasing the dose of IV STS has also been reported in the literature as a means of controlling side effects [41]. Careful monitoring of transient adverse effects is advised.

Several limitations of this study should be noted. First, the sample size was small, and there was a lack of data pertaining to pruritus risk factors and multiple concurrent treatments, which makes attributing the relief of symptoms to STS difficult. Second, we did not monitor changes in serum calcium, phosphorus, sodium, or other electrolytes after each dose, nor did we perform electrocardiography. Due to the short study period, we were also unable to study the effect of STS on the survival rate of HD patients. Third, there is no established system for exploring and documenting adverse drug reactions in the daily common practice of physicians and nurses in our dialysis center. We are concerned about adverse reactions to STS because some adverse reactions might not have been recorded in the medical records of these patients. Additionally, the high cost of STS in China (a 3.2 g dose given three times weekly costs approximately ¥800 per week) limits its use. Consequently, obtaining STS may be very difficult, even for hospitalized patients. Despite growing evidence of the efficacy of STS for the treatment of uremic pruritus, its administration for this indication is still considered an off-label, which limits the use of this drug.

To our knowledge, this is the first detailed report of the effectiveness and safety of the IV administration of STS for the treatment of uremic pruritus. Our study showed that STS is effective and safe for the treatment of uremic pruritus. Well-designed randomized controlled trials are needed to determine the efficacy, safety, optimum dosage, and duration of STS therapy for pruritus in HD patients.

## Abbreviations

HD: hemodialysis
STS: sodium thiosulfate
ESRD: end-stage renal disease
CUA: calcific uremic arteriolopathy
PTH: parathyroid hormone
VAS: visual analog scale
DPS: detailed pruritus score
hs-CRP: high-sensitivity C-reactive protein
AVF: arteriovenous fistula
